# Supplementary figures and images for: Identification of Auxin, Cytokinin, Transcription Factors, and Other Zygotic Embryogenesis-Related Genes in Persea americana: A Transcriptomic-Based Study
Source: Plants (Basel). 2025 Oct 27;14(21):3288. doi: 10.3390/plants14213288 (PMC12608710; doi:10.3390/plants14213288)

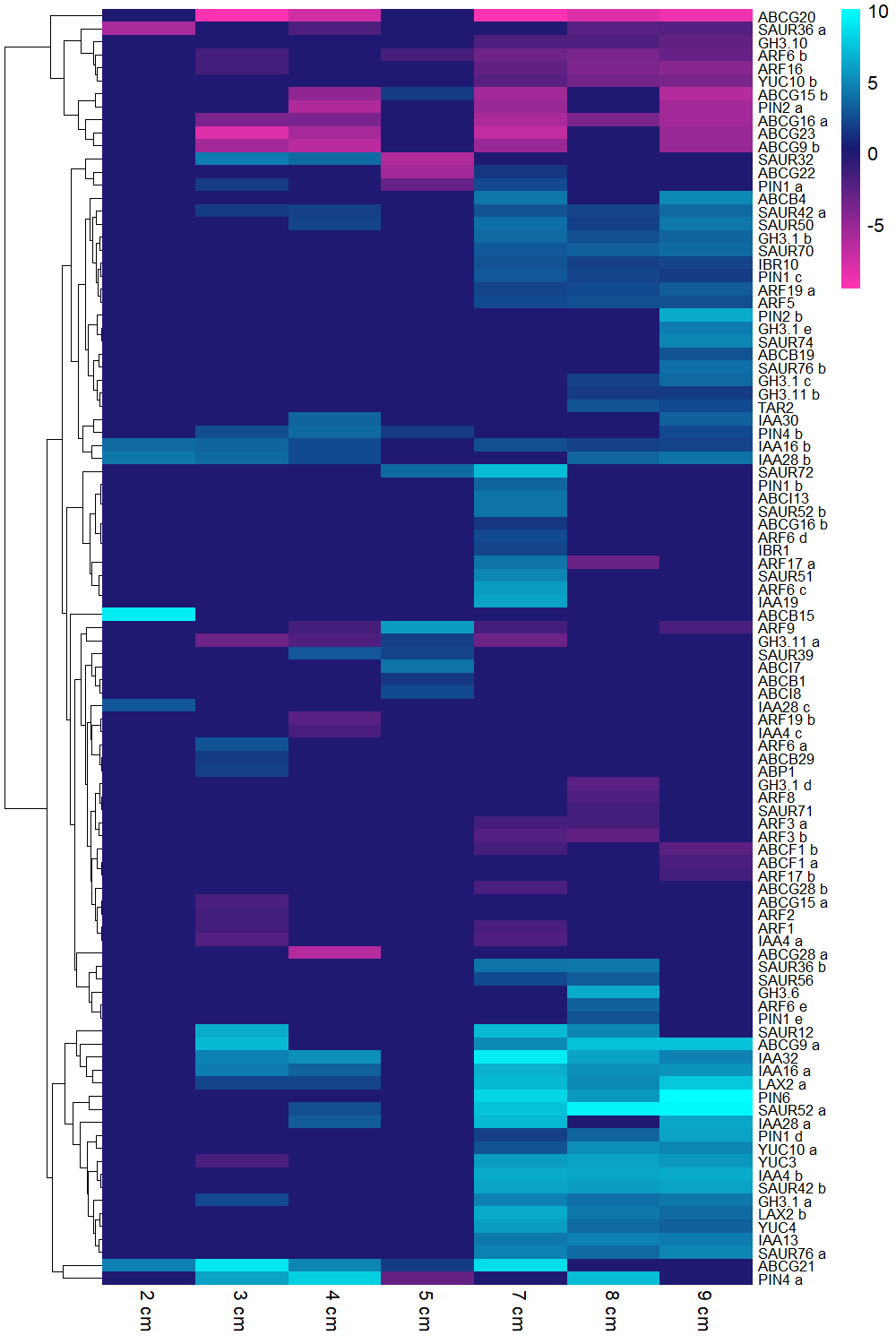

Supplement: Supplementary file 1 [file plants-14-03288-s001.zip › Figure S1.png]

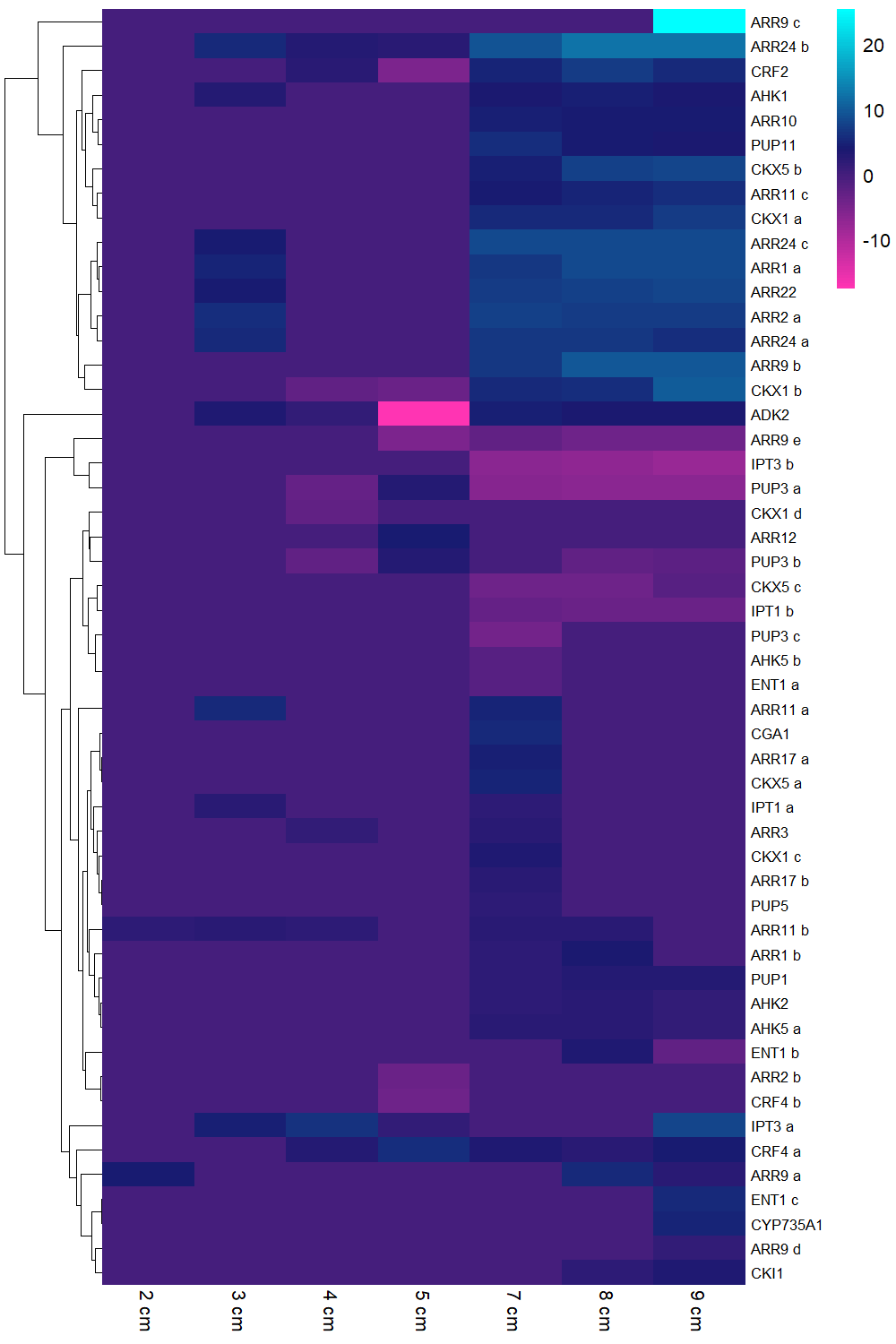

Supplement: Supplementary file 1 [file plants-14-03288-s001.zip › Figure S2.png]

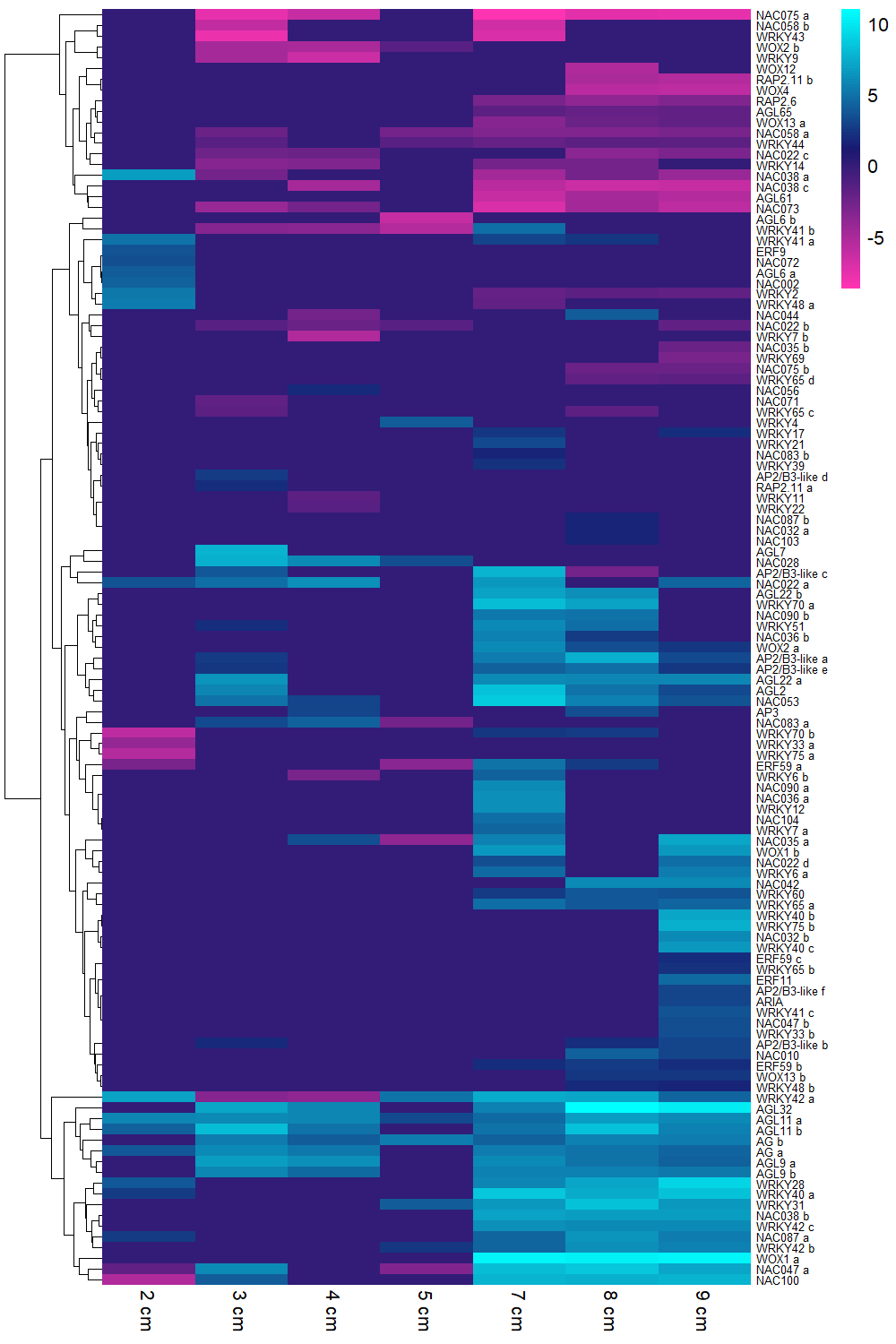

Supplement: Supplementary file 1 [file plants-14-03288-s001.zip › Figure S3.png]

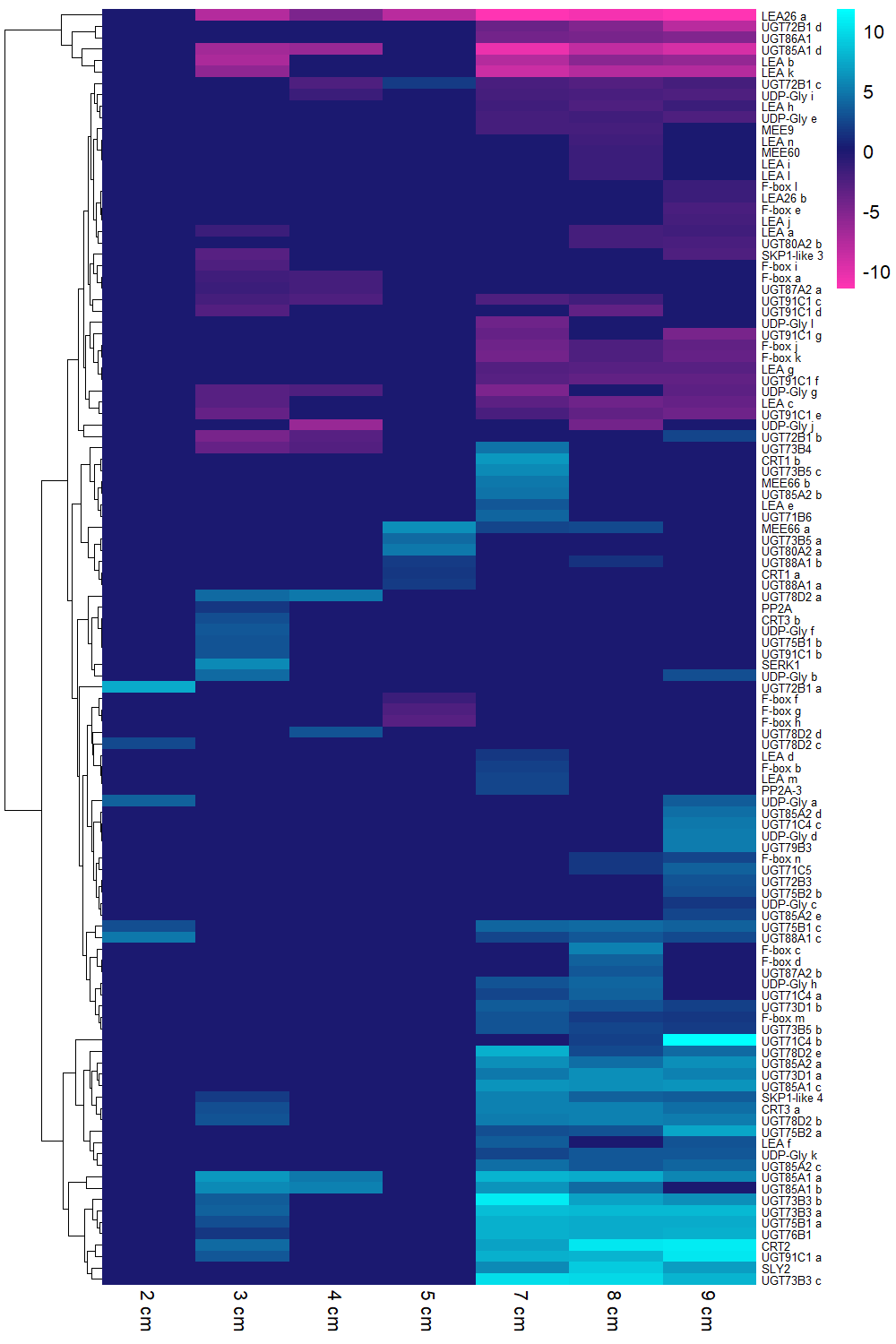

Supplement: Supplementary file 1 [file plants-14-03288-s001.zip › Figure S4.png]

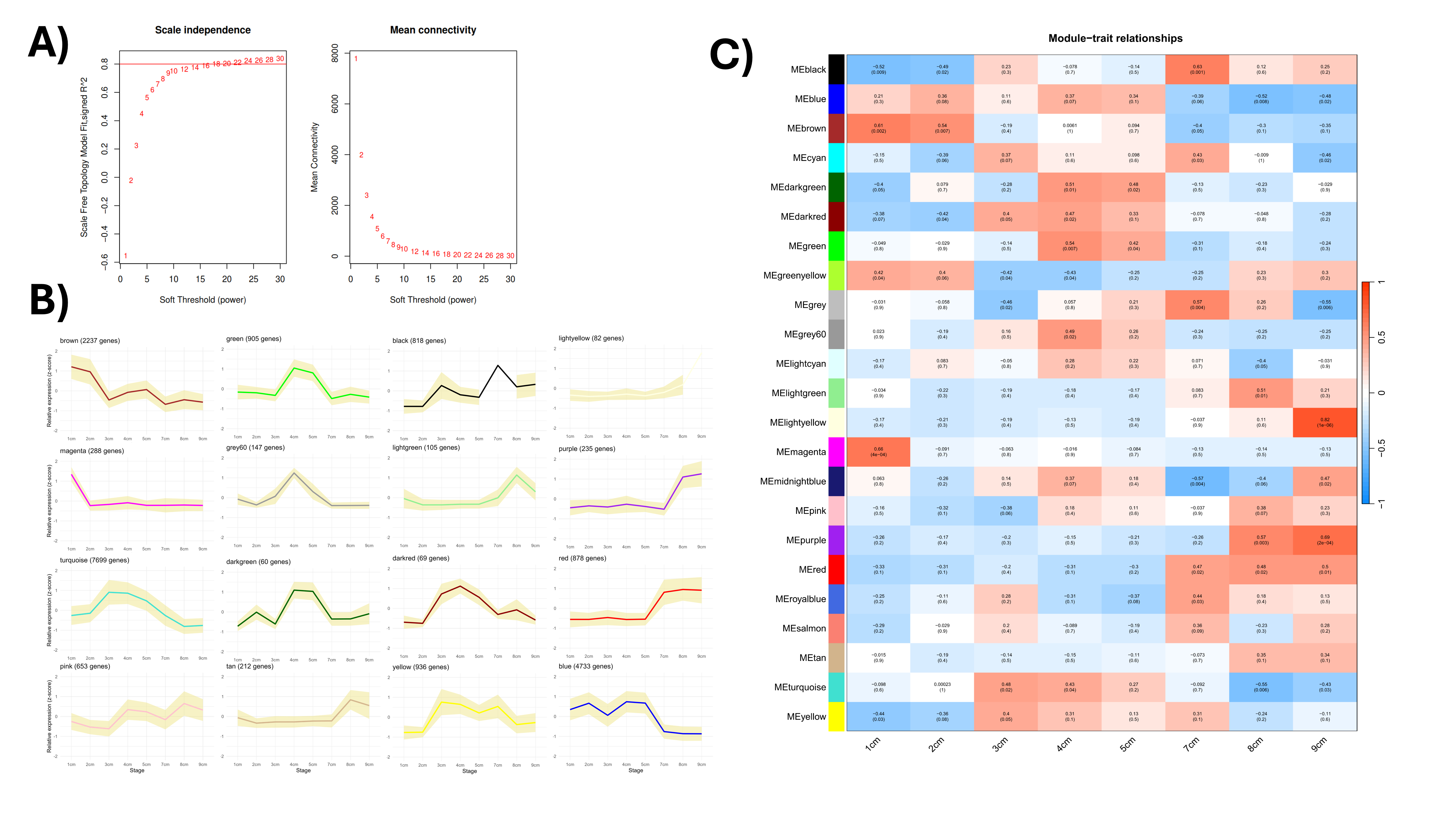

Supplement: Supplementary file 1 [file plants-14-03288-s001.zip › Figure S5.png]
